# Bazaars and Bankruptcy

**Published:** 1891-06-20

**Authors:** 


### Passing Topics.

### BAZAARS AND BANKRUPTCY.

It is only natural, we suppose, that the lay press, which did a good stroke of business in advertising and puffing the "Coming Race" Bazaar, should now make merry over the story of its failure, spiced as it is with the episode of the so-called "bogus" purses and the subsequent bankruptcy of its chief promoter, who, we fear, will look in vain for sympathy even among those who are most ready to regard with a lenient eye the excesses of zeal into which charity-workers are now and again betrayed.

For our part, we have not waited for failure to render us mindful of what we think to be due to the cause of charity. We have long held to the opinion that bazaars and lotteries, as customarily arranged, are a source of discredit and grave evil, and whatever good purpose they have served in earlier times, they are now become altogether unworthy to be enlisted in the cause of respectable institutions. Not only do they involve the speculative expenditure of a vast sum of money which it is certain no Committee is justified in risking, and which, whatever took place in the case under notice, is not likely to be risked by an ordinary promoter without the prospect of an adequate *quid pro quo* of some kind; but when the venture is at last fairly floated, financial success is made probable only by the employment of such arts, subterfuges, mis-statements, and misdoings generally, as under any other circumstances would be called by very ugly names.

We can imagine few things less defensible or more unpleasant and mischievous than the wholesale importunity and fibbing which is practised by the cloud of assistants, mostly very young ladies, who are sent forth equipped with but one desire—to get all they can out of any one who will consent to be fleeced. Of *bonâ fide* sales of goods at fair and reasonable figures there are none; the buyer who looked for them would be smiled at for a novice, and although we do not altogether admire the laboured facetiousness of the *Daily Telegraph*, which evidently has its own notions of taste as of language, we cannot deny there is plenty of truth in the comical description it has lately given of a Charity Bazaar, "where the distinction between *meum* and *tuum* is set at nought, and the fraudulent extraction of money from the pockets of visitors becomes an object not only undisguised, but openly and sometimes even boastfully proclaimed."

To admit even the partial accuracy of an indictment of this description is surely to condemn bazaars altogether. That there have been some good ones in old days, carried out with an ability and zeal which did not overlook the proprieties, we unhesitatingly admit. The building of churches, and many other irreproachable objects, have been substantially helped forward by efforts of the kind, and although we have sometimes wondered that the same divine who saw perdition in a game of whist or Pope Joan, could look with approval upon unlimited raffling, and that severe Evangelicals could apparently borrow Jesuitical principles for the nonce, we do not know that any great harm has been done. But Ice Carnivals, Coming Races, and the like have altered all that. These monster affairs, demoralizing as they are, have moreover been arranged usually in aid of very insignificant institutions, and it will not be astonishing if respectable and important charities henceforth decide to abandon bazaars in favour of efforts, less startling, perhaps, but more legitimate.

Agricola.

